# *NR3C1* Exon 1F Promoter Methylation and mRNA Expression in the Dorsolateral Prefrontal Cortex of Individuals Who Died by Suicide: A Post-Mortem Case–Control Study from Northern Mexico

**DOI:** 10.3390/brainsci16101059

**Published:** 2026-09-30

**Authors:** Yazmín M. Amador-Segovia, Daniel F. Ramos-Rosales, Víctor M. Ayala-García, Norma Urtiz-Estrada, Maribel Cervantes-Flores, Luz I. Valenzuela-García, Sandra I. Torres-Herrera, José M. Salas-Pacheco, Miguel A. Leyva López, Marcelo Barraza-Salas

**Affiliations:** 1 Facultad de Ciencias Químicas, Universidad Juárez del Estado de Durango, Durango P.C. 34120, Mexico; yazminamador22@gmail.com (Y.M.A.-S.); victor.ayala@ujed.mx (V.M.A.-G.); urtixnue@gmail.com (N.U.-E.); maribel.cervantes@ujed.mx (M.C.-F.); cp.angelleyva@gmail.com (M.A.L.L.); 2 Instituto de Investigación Científica, Universidad Juárez del Estado de Durango, Durango P.C. 34000, Mexico; daniel.ramos@ujed.mx (D.F.R.-R.); jose.salas@ujed.mx (J.M.S.-P.); 3 Centro de Investigación en Materiales Avanzados, S.C. (CIMAV), Unidad Durango, Durango P.C. 34147, Mexico; luz.valenzuela@cimav.edu.mx; 4 Facultad de Ciencias Forestales y Ambientales, Universidad Juárez del Estado de Durango, Durango P.C. 34120, Mexico; sandra.torres@ujed.mx

**Keywords:** suicide, *NR3C1*, DNA methylation, prefrontal cortex, epigenetic regulation

## Abstract

**Highlights:**

**What are the main findings?**

- Suicide decedents showed higher regional methylation within the assayed *NR3C1* exon 1F promoter region in BA9 than frequency-matched non-psychiatric controls (*p* = 0.00349; Cliff’s δ = 0.657).
- Total *NR3C1* mRNA expression was also higher in suicide decedents (*p* = 0.04412; Cliff’s δ = 0.467), although this finding should be considered preliminary.

**What are the implications of the main findings?**

- Regional methylation within the assayed exon 1F promoter region and total *NR3C1* mRNA expression were not significantly correlated and should be interpreted as separate group-level differences.
- Replication in larger, sex-balanced cohorts using CpG-, transcript-, and cell-type-resolved approaches is needed to clarify the biological significance of these findings.

**Abstract:**

Background: The glucocorticoid receptor gene (*NR3C1*) regulates hypothalamic–pituitary–adrenal (HPA) axis feedback. Evidence of its epigenetic and transcriptional alterations in suicide remains limited and inconsistent, particularly in underrepresented populations. Methods: We measured regional methylation within the assayed *NR3C1* exon 1F promoter region and total *NR3C1* mRNA expression in dorsolateral prefrontal cortex (BA9) from 13 suicide cases and 13 non-psychiatric controls from northern Mexico. Groups were frequency-matched by age, sex, and post-mortem interval. Methylation was assessed by methylation-sensitive restriction enzyme (MSRE) qPCR and expression by RT-qPCR. Group differences were tested using two-sided exact Mann–Whitney *U* tests; effect sizes were probability of superiority (PS) and Cliff’s δ. Correlation and male-only analyses were exploratory. All *p*-values were nominal. Results: Cases showed higher total *NR3C1* mRNA expression (U = 45, *p* = 0.04412; PS = 0.734; δ = 0.467) and regional exon 1F methylation (U = 29, *p* = 0.00349; PS = 0.828; δ = 0.657). The measures showed a non-significant positive correlation (ρ = 0.27, *p* = 0.179). In males, methylation remained higher in cases (*p* = 0.0147), whereas expression remained directionally higher but non-significant (*p* = 0.165). Conclusions: Individuals who died by suicide showed higher regional exon 1F methylation and *NR3C1* mRNA expression in BA9. The measures were not significantly correlated and should be interpreted as separate group-level differences, not evidence of direct methylation–expression coupling. The expression finding is preliminary. Given the small sample, limited clinical information, and comparison with non-psychiatric controls, the differences cannot be attributed specifically to suicide and require replication.

## 1. Introduction

Suicide is a major global public health concern, accounting for more than 720,000 deaths annually and ranking as the third leading cause of death among individuals aged 15–29 years worldwide [1]. In Mexico, suicide rates have steadily increased over the past decade, disproportionately affecting adolescents and young adults, thereby placing a growing burden on mental health care systems [2]. Although significant progress has been made in the diagnosis and pharmacological treatment of mood disorders, the biological mechanisms that drive suicidal behavior remain incompletely understood. This knowledge gap continues to hinder the development of effective, biologically informed prevention strategies.

Dysregulation of the hypothalamic–pituitary–adrenal (HPA) axis has emerged as a key pathophysiological feature in stress-related psychiatric conditions, including major depressive disorder and suicidal behavior [3,4]. Central to HPA axis regulation is the glucocorticoid receptor (GR), encoded by the *NR3C1* gene, which modulates negative feedback in response to circulating cortisol. Epigenetic alterations (particularly DNA methylation within promoter regions of *NR3C1*) can influence gene transcription and receptor sensitivity to glucocorticoids. Numerous studies have linked *NR3C1* promoter methylation to early-life adversity, trauma, and suicidality, highlighting its potential role as a molecular mediator of stress vulnerability [5,6].

Importantly, *NR3C1* regulation is highly region- and cell-type-specific within the brain. For instance, in hippocampal tissue from suicide victims with a history of childhood abuse, increased methylation of the exon 1F promoter of *NR3C1* was associated with decreased expression of GR-mRNA [7]. In a separate study of prefrontal cortex from suicide group, reduced expression of a transcript of *NR3C1* was reported [8].

The dorsolateral prefrontal cortex (dlPFC), including Brodmann area 9 (BA9), plays a central role in executive control, decision-making and cognitive appraisal of stress-domains that are critically implicated in suicidal behavior [9]. Rizavi et al. [6] previously examined *NR3C1* exon-1 methylation, hydroxymethylation, and transcript-specific expression in post-mortem BA9 from teenage suicide decedents, establishing an important precedent for the integrated study of epigenetic and transcriptional regulation of *NR3C1* in this region. However, evidence from independent populations and alternative analytical approaches remains limited.

The present study examined regional *NR3C1* exon 1F promoter methylation and total *NR3C1* mRNA expression in BA9 samples from individuals who died by suicide and non-psychiatric controls from Durango, northern Mexico. We hypothesized that suicide cases would show higher regional methylation and lower *NR3C1* expression. We also explored the relationship between these measurements. This study extends previous research by providing data from an independent northern Mexican cohort.

## 2. Methods

### 2.1. Study Subjects and Sample Collection

This retrospective case–control study included 13 suicide cases and 13 non-psychiatric controls. Controls were frequency-matched to cases at the group level by age, sex, and post-mortem interval (PMI); no individual case–control pairs were formed. All samples were obtained from the Forensic Medical Service (SEMEFO) of Durango, Mexico, under approval of the Research Ethics Committee of Hospital General de Durango (registration number CONBIOETICA-10-CEI-001-20170421). Written informed consent was obtained from the next of kin for each subject prior to sample collection. During autopsy, the attending forensic pathologist anatomically identified and dissected BA9 from the left dorsolateral prefrontal cortex. Samples comprised cortical gray matter and adjacent white matter and were processed as bulk tissue. The tissue was divided into small fragments, immersed in RNAlater™ stabilization solution (Invitrogen, Thermo Fisher Scientific, Waltham, MA, USA), and stored at −80 °C until RNA and DNA extraction.

Inclusion criteria for suicide cases comprised: (i) confirmed cause of death by suicide according to medico-legal autopsy and forensic reports, (ii) absence of chronic systemic or neurological diseases, and (iii) PMI ≤ 16 h. Controls were individuals who died suddenly from non-neurological, non-suicidal causes (e.g., traffic accidents or other trauma). Information on psychiatric diagnoses and drug use in potential controls was obtained through interviews with their next of kin. Potential controls were excluded when relatives reported a history of psychiatric diagnosis or drug use. Accordingly, the control group comprised individuals with no such history reported by family members, although this information was not independently verified through medical records or a standardized psychological autopsy. Both groups included 10 males and 3 females.

### 2.2. NR3C1 Promoter Methylation Analysis

Genomic DNA was extracted using the Wizard^®^ Genomic DNA Purification Kit (Promega, Madison, WI, USA; Cat. No. A1120), following the manufacturer’s protocol. DNA concentration and purity were determined spectrophotometrically (NanoDrop 2000, Thermo Fisher Scientific, Waltham, MA, USA), and integrity was verified by 1% agarose gel electrophoresis. Samples with A260/A280 ratios between 1.8 and 2.0 were considered acceptable for downstream analysis.

DNA methylation at the *NR3C1* promoter (exon 1F region) was quantified using the OneStep™ qMethyl PCR Kit (Zymo Research, Irvine, CA, USA; Cat. No. D5312), following the manufacturer’s protocol.

A single primer pair was designed in silico using MethPrimer to amplify a 264 bp fragment spanning part of *NR3C1* exon 1F and its immediately upstream promoter region (GRCh38/hg38, chr5:143,404,220–143,404,483; reverse strand), containing 29 CpG sites within the amplicon. Primer sequences were forward 5′-CAACTTCTCTCCCAGTGCGAGAG-3′ and reverse 5′-CGAGTTGCGTGAAGTGTGTCAC-3′. The amplicon contains 16 recognition motifs for the MSREs included in the enzyme mixture: four CCGG, three GCGC, six CCGC, and three GCGG motifs. Therefore, the assay provides a regional methylation estimate based on combined protection from digestion at these sites.

Each reaction was performed in a total volume of 20 µL, containing 10–20 ng of genomic DNA, qMethyl Master Mix, qMethyl Enzyme Mix, and 0.3 µM of each primer. Both reactions were incubated at 37 °C for 2 h. Amplification was carried out using a QuantStudio™ 3 Real-Time PCR System (Applied Biosystems, Thermo Fisher Scientific, Waltham, MA, USA) under the following thermal cycling conditions: initial denaturation at 95 °C for 10 min, followed by 40 cycles of 95 °C for 15 s and 60 °C for 1 min.

The Human Methylated and Non-methylated DNA Standards supplied with the kit, together with the corresponding qMethyl control primers, were used as positive and negative controls to verify MSRE activity and assay performance. According to the manufacturer, the non-methylated standard retains approximately 5% residual methylation.

For each sample, two parallel reactions were performed: a Test Reaction containing the methylation-sensitive enzyme mix and a Reference Reaction without enzyme. Methylation percentage was calculated using the following equation:

*Methylation* (%) = 100 × 2^−Δ*Ct*^

Δ*Ct* = *Ct_Test_* − *Ct_Reference_*

Higher methylation at the MSRE recognition sites protects the target DNA from enzymatic digestion. Consequently, the Ct of the Test Reaction approaches that of the Reference Reaction, resulting in a smaller ΔCt and a higher estimated methylation percentage. All reactions were performed in triplicate. No-template controls were included to detect potential contamination, and melting-curve analysis was used to confirm amplification specificity.

### 2.3. NR3C1 Gene Expression Analysis

Total RNA was extracted from dlPFC samples using the Direct-zol™ RNA Miniprep Plus Kit (Zymo Research, Irvine, CA, USA; Cat. No. R2072), according to the manufacturer’s instructions.

Complementary DNA was synthesized using 1 µg of total RNA with the High-Capacity cDNA Reverse Transcription Kit (Applied Biosystems, Thermo Fisher Scientific; Cat. No. 4368814), following the standard protocol (25 °C for 10 min, 37 °C for 120 min, and 85 °C for 5 min).

Quantitative real-time PCR was performed to assess *NR3C1* mRNA expression levels using TaqMan^®^ Gene Expression Assays (Applied Biosystems) specific for *NR3C1* (Assay ID: Hs00353740_m1) and the endogenous control *B2M* (Assay ID: Hs00187842_m1). *B2M* was selected based on its previous use as a reference gene in RT-qPCR studies of post-mortem prefrontal cortex, including a suicide cohort in which it showed consistent expression across samples [10] and psychiatric case–control cohorts [11,12]. Each reaction was run in triplicate in 20 µL volumes using TaqMan™ Universal PCR Master Mix (Applied Biosystems; Cat. No. 4304437) on a QuantStudio™ 3 Real-Time PCR System under standard cycling conditions: 50 °C for 2 min, 95 °C for 10 min, followed by 40 cycles of 95 °C for 15 s and 60 °C for 1 min. Relative expression was calculated using the 2^−ΔΔ*Ct*^ method, where Δ*Ct* = *Ct*_NR3C1_ − *Ct*_B2M_ and ΔΔ*Ct* = Δ*Ct* sample − Δ*Ct* calibrator. One control sample was used as the calibrator and was assigned a relative expression value of 1. No-template and no-reverse-transcription controls were included to detect contamination and genomic DNA amplification, respectively.

### 2.4. Statistical Analysis

Statistical analyses were performed using IBM SPSS Statistics version 25.0 (IBM Corp., Armonk, NY, USA) and R version 4.5.0. Continuous variables were summarized as median (Q1–Q3) and compared using two-sided exact permutation Mann–Whitney *U* tests. Because matching was performed at the group level and no individual case–control pairs were formed, observations were treated as independent. Sex distribution was compared using Fisher’s exact test. Effect sizes were expressed as the probability of superiority (PS) and Cliff’s δ, with positive values indicating higher measurements in suicide cases. The relationship between *NR3C1* methylation and expression was explored using Spearman’s rank correlation. Given the limited number of females, molecular comparisons were repeated exploratorily in males only. Suicide method was summarized descriptively without formal between-method inference because three of the four categories contained only one individual. No formal correction for multiple comparisons was applied. A two-sided nominal *p* < 0.05 was considered statistically significant.

An effect-size sensitivity analysis was conducted using the *pwrss* v.1.2.0 in R and the Noether approximation for the Wilcoxon-Mann–Whitney test. With 13 individuals per group and α = 0.05, 80% power required a PS ≥ 0.82, equivalent to Cliff’s δ ≥ 0.63.

## 3. Results

### 3.1. Cohort Characteristics and Demographics and Causes of Death

A total of 13 individuals who died by suicide and 13 non-psychiatric controls were included in the study. Groups were matched for age, sex, and PMI. Each group consisted of 10 males and 3 females. No statistically significant differences were found between groups for age, sex distribution, or PMI (Table 1).

Among individuals who died by suicide, asphyxia was the predominant method (*n* = 10; 76.9%). Poisoning, gunshot wounds, and drug overdose each accounted for one case (*n* = 1; 7.7%). In the control group, causes of death included traffic-related accidents (*n* = 7; 53.8%), multiple trauma (*n* = 4; 30.8%), drowning (*n* = 1; 7.7%), and gunshot wounds (*n* = 1; 7.7%).

### 3.2. Prefrontal Cortex Methylation-Expression Profile

**Figure 1:**
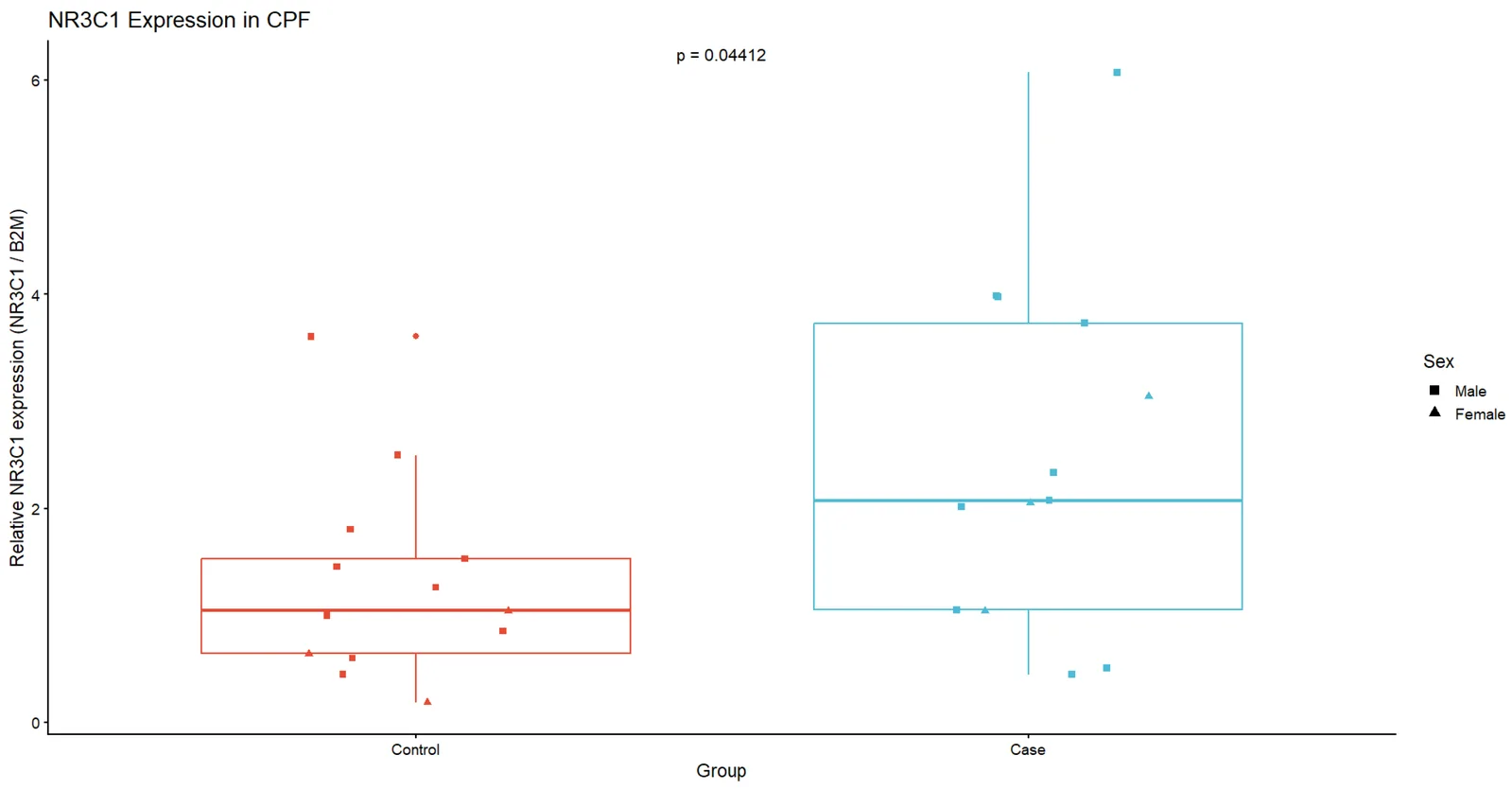
Increased *NR3C1* mRNA Expression in the dlPFC of Suicide Decedents. Relative total *NR3C1* mRNA expression, normalized to *B2M*, in suicide cases (*n* = 13) and non-psychiatric controls (*n* = 13). Boxes represent the median and interquartile range, and whiskers extend to the most extreme values within 1.5 times the interquartile range. Individual observations are overlaid; squares denote males and triangles denote females. Expression was higher in suicide cases (two-sided exact Mann–Whitney *U* = 45, nominal *p* = 0.04412).

**Figure 2:**
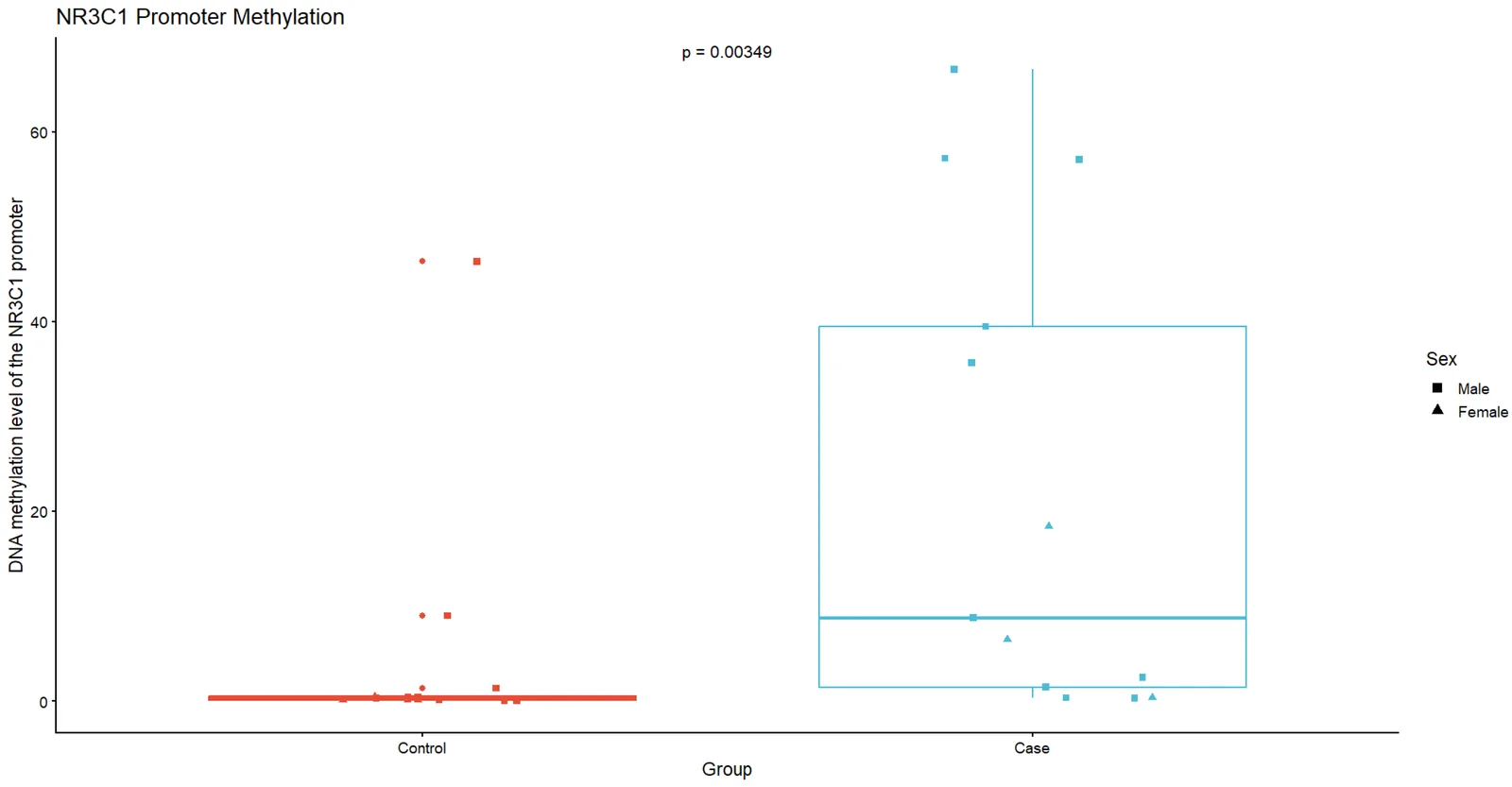
Increased *NR3C1* Promoter Methylation in the dlPFC of Suicide Decedents. Regional methylation estimates within the assayed *NR3C1* exon 1F promoter region in suicide cases (*n* = 13) and non-psychiatric controls (*n* = 13). Boxes represent the median and interquartile range, and whiskers extend to the most extreme values within 1.5 times the interquartile range. Individual observations are overlaid; squares denote males and triangles denote females. Regional methylation was higher in suicide cases (two-sided exact Mann–Whitney *U* = 29, nominal *p* = 0.00349).

Prefrontal cortex samples from suicide decedents exhibited significantly higher *NR3C1* mRNA expression compared with non-psychiatric controls (*U* = 45, *p* = 0.04412, Figure 1). In parallel, DNA methylation at the *NR3C1* promoter region (exon 1F) was also significantly higher in suicide cases (*U* = 29, *p* = 0.00349; Figure 2).

**Figure 3:**
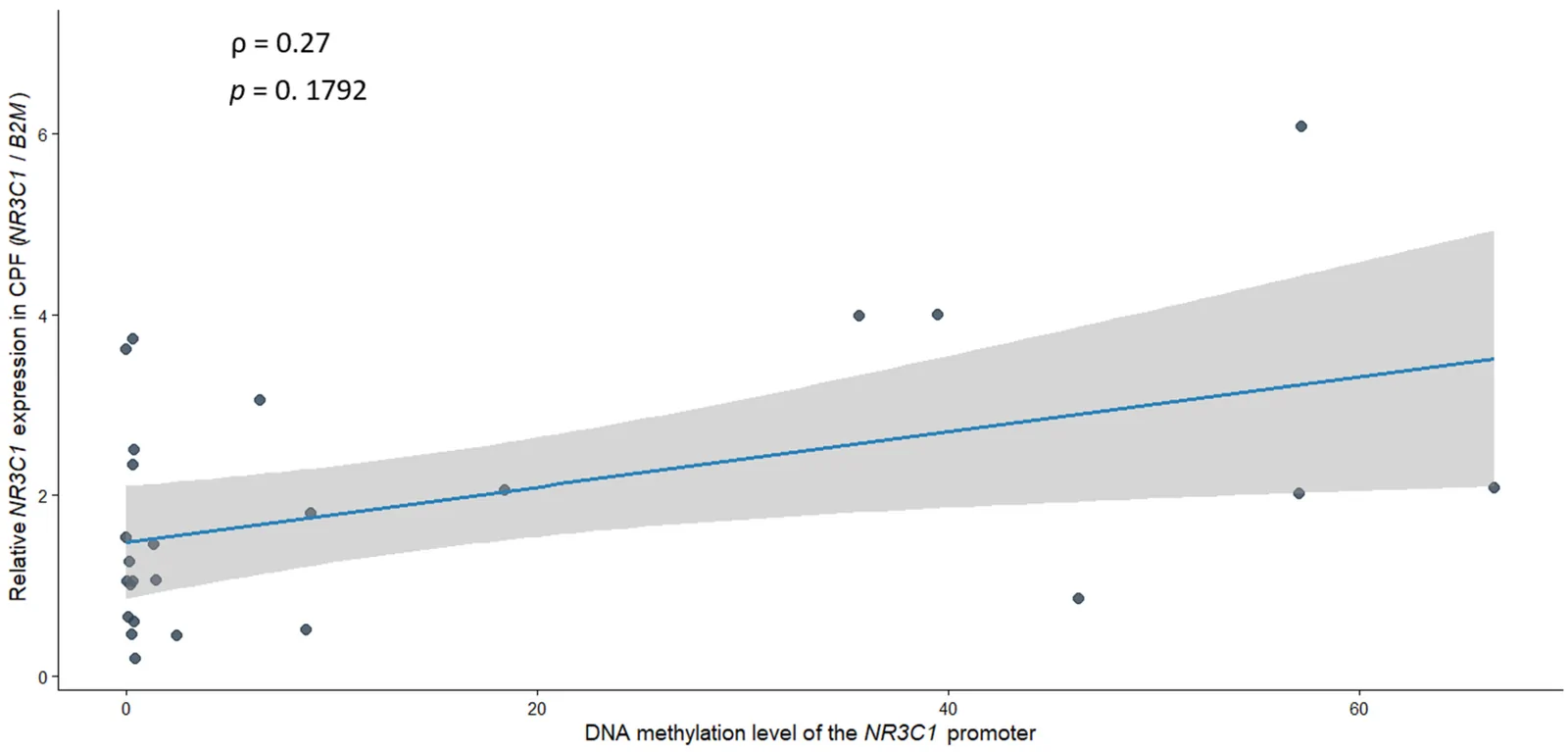
Correlation Between *NR3C1* Methylation and Expression in the dlPFC. Scatter plot showing the relationship between regional methylation within the assayed *NR3C1* exon 1F promoter region and relative total *NR3C1* mRNA expression normalized to *B2M* in suicide cases and controls. Each point represents one individual sample. The blue line shows the fitted linear trend, and the gray shading represents its 95% confidence interval; both are included only to illustrate the overall direction of the association. Spearman’s correlation showed a non-significant positive association (ρ = 0.27, *p* = 0.1792).

The exploratory Spearman analysis showed a non-significant positive correlation between *NR3C1* promoter methylation and mRNA expression, although this relationship was not statistically significant (ρ = 0.27, *p* = 0.1792; Figure 3).

### 3.3. Effect Sizes and Sensitivity Analysis

The estimated probability that a randomly selected individual from the suicide group had a higher *NR3C1* expression value than a randomly selected control was 0.734, corresponding to a Cliff’s δ of 0.467. For regional exon 1F promoter methylation, PS was 0.828, corresponding to a Cliff’s δ of 0.657.

The sensitivity analysis indicated that, with 13 individuals per group and a two-sided α = 0.05, the minimum effect detectable with 80% power in the observed direction corresponded to PS = 0.817, equivalent to a Cliff’s δ of 0.634. Thus, the available sample was primarily sensitive to large case–control differences.

### 3.4. Exploratory Male-Only Analyses

Given the limited number of females (*n* = 3 per group), female-specific comparisons and group-by-sex interaction analyses were not performed. The primary comparisons were therefore repeated exploratorily in males (*n* = 10 per group). *NR3C1* expression remained higher in suicide cases than in controls, although the difference was not statistically significant (*p* = 0.165), whereas promoter methylation remained significantly higher in cases (*p* = 0.0147; Supplementary Figures S1 and S2). A non-significant positive association was observed between regional exon 1F methylation and total *NR3C1* mRNA expression in males (Spearman’s ρ = 0.215, *p* = 0.363; Supplementary Figure S3).

### 3.5. Exploratory Analyses by Suicide Method

*NR3C1* expression and promoter methylation were descriptively examined according to suicide method. Asphyxia accounted for 10 cases, whereas poisoning, gunshot wounds, and drug overdose were each represented by one case. Given the small and markedly unbalanced subgroups, formal between-method comparisons were not performed. Individual values are shown in Supplementary Figures S1–S3.

## 4. Discussion

In the dorsolateral prefrontal cortex (BA9), individuals who died by suicide showed higher regional methylation within the *NR3C1* exon 1F promoter and higher total *NR3C1* mRNA expression than non-psychiatric controls. The increase in *NR3C1* mRNA expression should be considered preliminary. Methylation and expression were not significantly correlated and should therefore be interpreted as separate group-level differences. Because asphyxia was the predominant method of suicide, a contribution of hypoxia or other method-related agonal effects cannot be excluded, although the present design does not allow their influence to be determined.

Within the glucocorticoid hypothesis of suicide, impaired HPA axis feedback has been associated with reduced glucocorticoid receptor (GR/*NR3C1*) signaling. In post-mortem hippocampal tissue, McGowan et al. [7] reported increased *NR3C1* exon 1F promoter methylation and reduced total and exon 1F-specific expression in individuals who died by suicide with a history of childhood abuse. Rizavi et al. [6] examined BA9 and the hippocampus in adolescents who died by suicide. Lower GR-1F transcript expression was observed in BA9 but not in the hippocampus. Their methylation analyses focused on BA9, where a region overlapping the exon 1F promoter showed increased 5mC and decreased 5hmC. Consistent with the methylation component of our a priori hypothesis, regional exon 1F methylation was higher in suicide cases. The expression result, however, was in the opposite direction from that hypothesized: total *NR3C1* mRNA expression was higher, rather than lower, in suicide cases. The basis of this unexpected finding remains unclear because exon 1F-specific transcripts, GR protein levels, and receptor function were not measured.

One factor that may contribute to this unexpected combination of findings is the complex 5′ regulatory architecture of *NR3C1*, which includes multiple alternative untranslated first exons controlled by distinct promoters. In the adult human dlPFC, transcripts containing exons 1B, 1C, and 1H are strongly expressed, whereas the exon 1F transcript is comparatively less abundant [13]. Because the present assay quantified total *NR3C1* expression rather than promoter-specific transcripts, methylation confined to the exon 1F region may not parallel total mRNA abundance. Differences across studies may likewise reflect the specific promoters and transcripts examined. In alcohol use disorder, increased methylation within the exon 1H region of the prefrontal cortex has been associated with reduced *NR3C1* mRNA and protein levels [14], further illustrating promoter-specific heterogeneity in *NR3C1* regulation.

Second, the use of bulk BA9 tissue limits the cellular interpretation of the findings. Both methylation and expression measurements represent aggregate signals from neurons and multiple glial populations, which can exhibit distinct molecular profiles. Therefore, it cannot be determined whether the observed methylation and expression differences originated in the same cell populations or whether variation in cellular composition contributed to the apparent dissociation between these measures. Cell-type-resolved studies will be required to evaluate this possibility [15,16].

Third, *NR3C1* regulation may involve molecular processes not captured by the targeted methylation assay. More broadly, gene transcription is influenced by three-dimensional chromatin architecture and distal enhancer-promoter interactions [17]; however, these mechanisms were not examined in the present study, and their contribution to *NR3C1* expression in BA9 remains unknown. Furthermore, *NR3C1* mRNA abundance does not necessarily reflect GR protein abundance or functional activity. The co-chaperone FKBP51, encoded by FKBP5, can reduce GR-dependent transcriptional activity and delay receptor nuclear translocation without altering GR protein levels [18].

Previous studies of BA9 have reported reduced GR expression and increased methylation at several *NR3C1* exon-1 regulatory regions in individuals who died by suicide [6,19]. In contrast, we found higher total *NR3C1* mRNA expression alongside higher regional exon 1F methylation. These differences should be interpreted cautiously because the studies examined different populations, regulatory regions, and transcript targets and used different methods to assess methylation. Transcriptomic reviews have also reported variation in the direction and magnitude of HPA-axis-related changes across cortical regions [20]. Whether these discrepancies reflect biological context, methodological differences, or both remains unclear. Moreover, the higher mRNA expression observed here cannot be taken as evidence of increased GR protein abundance or receptor signaling.

Studies of psychiatric disorders further illustrate the complexity of *NR3C1* transcriptional regulation. Reduced expression of specific exon-1 transcripts and increased expression of truncated GR isoforms have been reported in schizophrenia and mood disorders [21,22]. Altered promoter usage without substantial changes in methylation has also been described in major depression [23]. The consequences of altered *NR3C1* expression may vary according to the transcript or isoform examined, as well as the biological and developmental context [24]. Our findings add to this literature by documenting higher total *NR3C1* mRNA expression alongside higher regional exon 1F methylation. However, they do not establish corresponding changes in GR protein abundance or receptor function.

In the male-only analysis, promoter methylation remained higher in suicide cases, whereas the expression difference remained in the same direction but was no longer statistically significant. The persistence of the methylation difference is broadly consistent with previous male-only post-mortem studies reporting alterations in *NR3C1* methylation among suicide cases, although the affected brain regions, clinical context, and direction of the molecular changes have varied across studies [7,25]. However, this finding does not establish a sex-specific effect. Given the inclusion of only three females per group, group-by-sex interactions could not be evaluated, and the loss of statistical significance for expression in the male-only analysis may reflect reduced precision rather than a genuine difference between sexes.

These findings suggest that methylation measurements restricted to a single *NR3C1* region may not capture the complexity of GR regulation. Future studies integrating promoter-resolved methylation, transcript-specific expression, GR protein levels, FKBP5, functional HPA-axis measures, and corresponding assessments in accessible peripheral tissues could clarify the biological and translational significance of these alterations. However, their clinical and predictive utility remains to be established.

### Limitations and Future Directions

Several limitations constrain the interpretability and generalizability of this study. First, the small sample size (13 suicide cases and 13 controls) resulted in imprecise effect-size estimates and limited power to detect anything other than large between-group differences. The sensitivity analysis indicated that the available sample had 80% power only to detect large effects (probability of superiority ≥ 0.82; Cliff’s δ ≥ 0.63). The methylation difference showed a large effect (δ = 0.657), although its estimate remains imprecise and requires replication. In contrast, the expression effect was moderate (δ = 0.467), and its nominal *p*-value was close to the significance threshold; this finding should therefore be considered preliminary and hypothesis-generating.

Second, the retrospective post-mortem design limited clinical characterization. Detailed psychiatric, medication, substance-use, and psychosocial information was unavailable for the suicide cases. Control histories were obtained from next of kin but were not independently verified through medical records or a standardized psychological autopsy. Residual clinical confounding therefore cannot be excluded. Brain pH and RNA integrity measurements were unavailable. Method-of-death effects also could not be evaluated because 10 of the 13 cases died by asphyxia and each remaining method occurred only once. Hypoxic or other agonal effects may therefore have contributed to the observed differences.

Third, all measurements were derived from bulk tissue in BA9, a region comprising a heterogeneous mix of neurons, glial cells, and other populations. Without cell-type-specific resolution, the source of the observed epigenetic and transcriptional changes remains ambiguous, potentially masking divergent effects within specific cellular compartments.

Fourth, technical limitations affect the molecular resolution of the study. Methylation was assessed using a MSRE-qPCR assay targeting a single amplicon within the exon 1F region. The resulting value is a regional estimate based on protection from digestion at the 16 MSRE recognition sites and does not represent the arithmetic mean of methylation across all CpGs within the amplicon. This approach also does not resolve individual CpG sites, distinguish 5-methylcytosine from 5-hydroxymethylcytosine, or assess alternative promoters. Similarly, *NR3C1* expression was measured using a single TaqMan assay without isoform discrimination and normalized to *B2M*, whose stability was supported by previous post-mortem prefrontal cortex studies but was not independently evaluated in the present cohort. These constraints preclude detailed insights into promoter-specific or isoform-dependent regulation.

Fifth, the analysis was restricted to BA9; therefore, the findings cannot be generalized to other cortical or limbic regions implicated in suicide, including BA46, the orbitofrontal and anterior cingulate cortices, hippocampus, and amygdala. In addition, the absence of blood or saliva samples precluded assessment of central-peripheral concordance in *NR3C1* methylation and expression.

Sixth, the small number of females precluded reliable sex-specific comparisons and formal assessment of group-by-sex interactions; consequently, the male-only findings should be considered exploratory.

Finally, the cross-sectional nature of post-mortem data prevents causal inference. It remains unclear whether the epigenetic and expression patterns observed reflect pre-existing vulnerability, consequences of chronic stress or psychopathology, treatment effects, or a combination thereof.

Future studies should address these limitations by employing larger, well-characterized cohorts with detailed clinical histories and comprehensive metadata, including toxicological screens and RNA quality indicators. At the molecular level, high-resolution approaches (such as bisulfite sequencing, hydroxymethylation profiling, and long-read RNA sequencing) are needed to map *NR3C1* regulation with promoter and isoform specificity.

## 5. Conclusions

Individuals who died by suicide showed higher regional methylation within the *NR3C1* exon 1F promoter and higher total *NR3C1* mRNA expression in dlPFC than matched non-psychiatric controls. Because methylation and expression were not significantly correlated, these findings should be interpreted as parallel group-level differences rather than evidence that methylation within the assayed region directly increases transcription. The expression finding should be considered preliminary and does not establish increased GR protein abundance or receptor signaling.

These findings extend previous research by providing molecular data from an independent and underrepresented northern Mexican post-mortem cohort. However, potential contributions from method-related agonal effects and unmeasured psychiatric or clinical factors cannot be excluded. Replication in larger, clinically characterized cohorts using cell-type- and promoter-resolved methylation, transcript-specific expression, and GR protein measurements will be necessary to clarify the biological significance of this pattern.

## Figures and Tables

**Table 1:** Characteristics of suicide cases and controls.

Variable	Case (*n* = 13)	Control (*n* = 13)	*p*-Value
Sex (Male/Female)	10 (76.9%)/3 (23.1%)	10 (76.9%)/3 (23.1%)	1.000 †
Age (years)	26 (22–47)	28 (23–49)	0.714 *
PMI (hours)	4.0 (3.0–4.0)	3.65 (3.0–4.0)	0.539 *

Quantitative variables are presented as median (Q1–Q3). PMI, post-mortem interval. * Two-sided exact permutation Mann–Whitney *U* test; † Fisher’s exact test.

## Data Availability

The anonymized dataset generated and analyzed during the current study is publicly available in the Figshare repository at https://doi.org/10.6084/m9.figshare.33273873.

## Supplementary Materials

The following supporting information can be downloaded at https://www.mdpi.com/article/10.3390/brainsci16101059/s1, Figure S1: ***NR3C1* expression in dlPFC (males only).** Box plot showing relative *NR3C1* mRNA expression in dlPFC (Brodmann area 9), normalized to *B2M*, in suicide cases and non-psychiatric controls restricted to males (n ≈ 10 per group); Figure S2: ***NR3C1* promoter methylation in dlPFC (males only).** Box plot showing the percentage of DNA methylation at the *NR3C1* exon 1F promoter region in dlPFC (Brodmann area 9) samples from suicide cases and non-psychiatric controls restricted to males (n ≈ 10 per group); Figure S3: **Correlation between *NR3C1* promoter methylation and expression in dlPFC (males only).** Scatter plot depicting the association between *NR3C1* exon 1F promoter methylation (%) and *NR3C1* mRNA expression in dlPFC (Brodmann area 9) restricted to males (n = 20).

## Abbreviations

5mC	5-Methylcytosine
BA9	Brodmann area 9
*B2M*	Beta-2-microglobulin
cDNA	Complementary DNA
Ct	Cycle threshold
dlPFC	Dorsolateral prefrontal cortex
DNA	Deoxyribonucleic acid
GR	Glucocorticoid receptor
GRCh38/hg38	Genome Reference Consortium Human Build 38
HPA	Hypothalamic–pituitary–adrenal
mRNA	Messenger RNA
MSRE	Methylation-sensitive restriction enzyme
*NR3C1*	Nuclear receptor subfamily 3 group C member 1
PCR	Polymerase chain reaction
PMI	Post-mortem interval
PS	Probability of superiority
qPCR	Quantitative polymerase chain reaction
RNA	Ribonucleic acid
RT-qPCR	Reverse-transcription quantitative polymerase chain reaction
SEMEFO	Servicio Médico Forense (Forensic Medical Service)
