# Supplementary material for: NR3C1 Exon 1F Promoter Methylation and mRNA Expression in the Dorsolateral Prefrontal Cortex of Individuals Who Died by Suicide: A Post-Mortem Case–Control Study from Northern Mexico
Source: Brain Sci. 2026 Sep 30;16(10):1059. doi: 10.3390/brainsci16101059 (PMC13702240; doi:10.3390/brainsci16101059)
Supplement: Supplementary file 1 [file brainsci-16-01059-s001.zip › brainsci-4525896-supplementary.pdf]

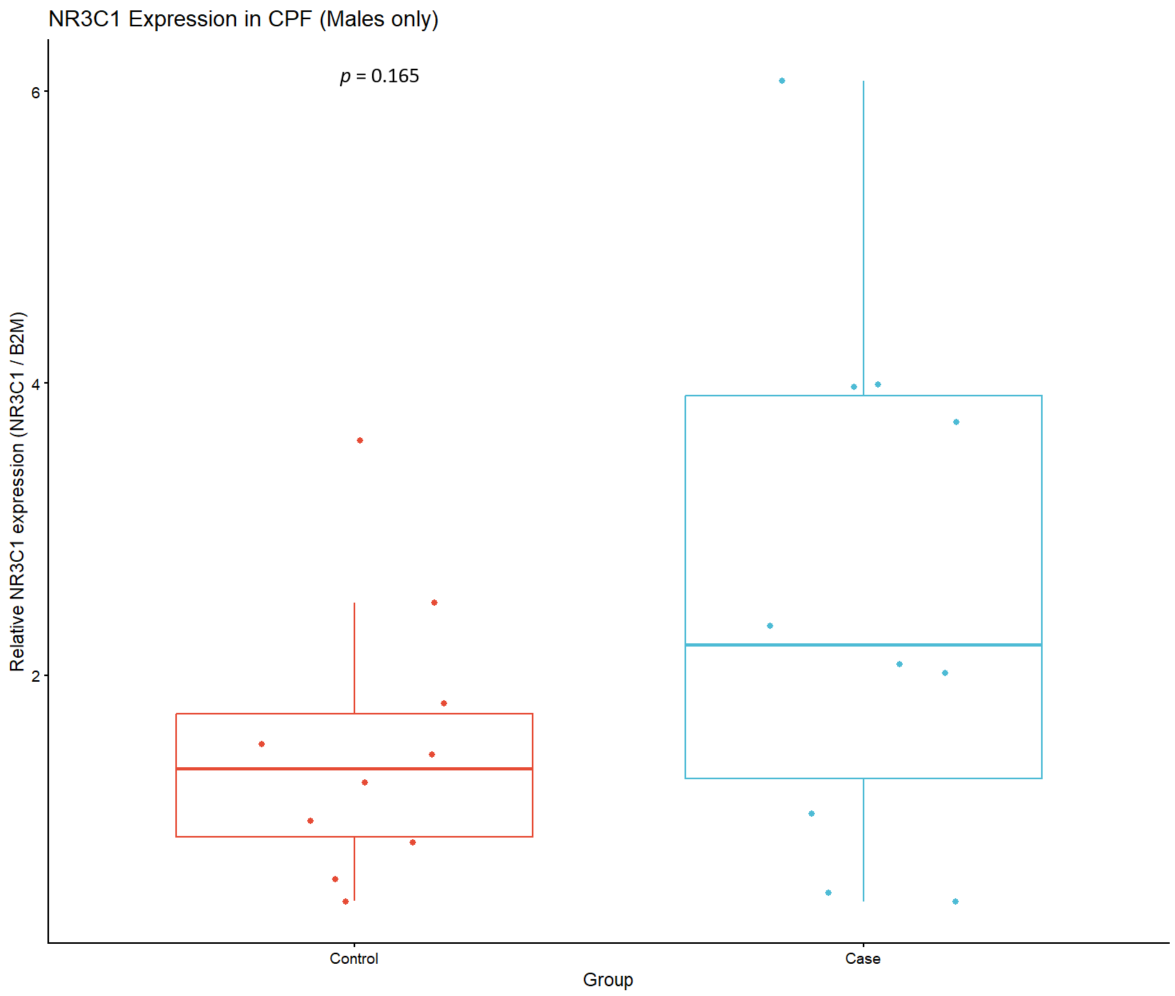

Supplementary Figure S1. ***NR3C1* expression in dlPFC (males only).** Box plot showing relative *NR3C1* mRNA expression in dlPFC (Brodmann area 9), normalized to *B2M*, in suicide cases and non-psychiatric controls restricted to males (n ≈ 10 per group).

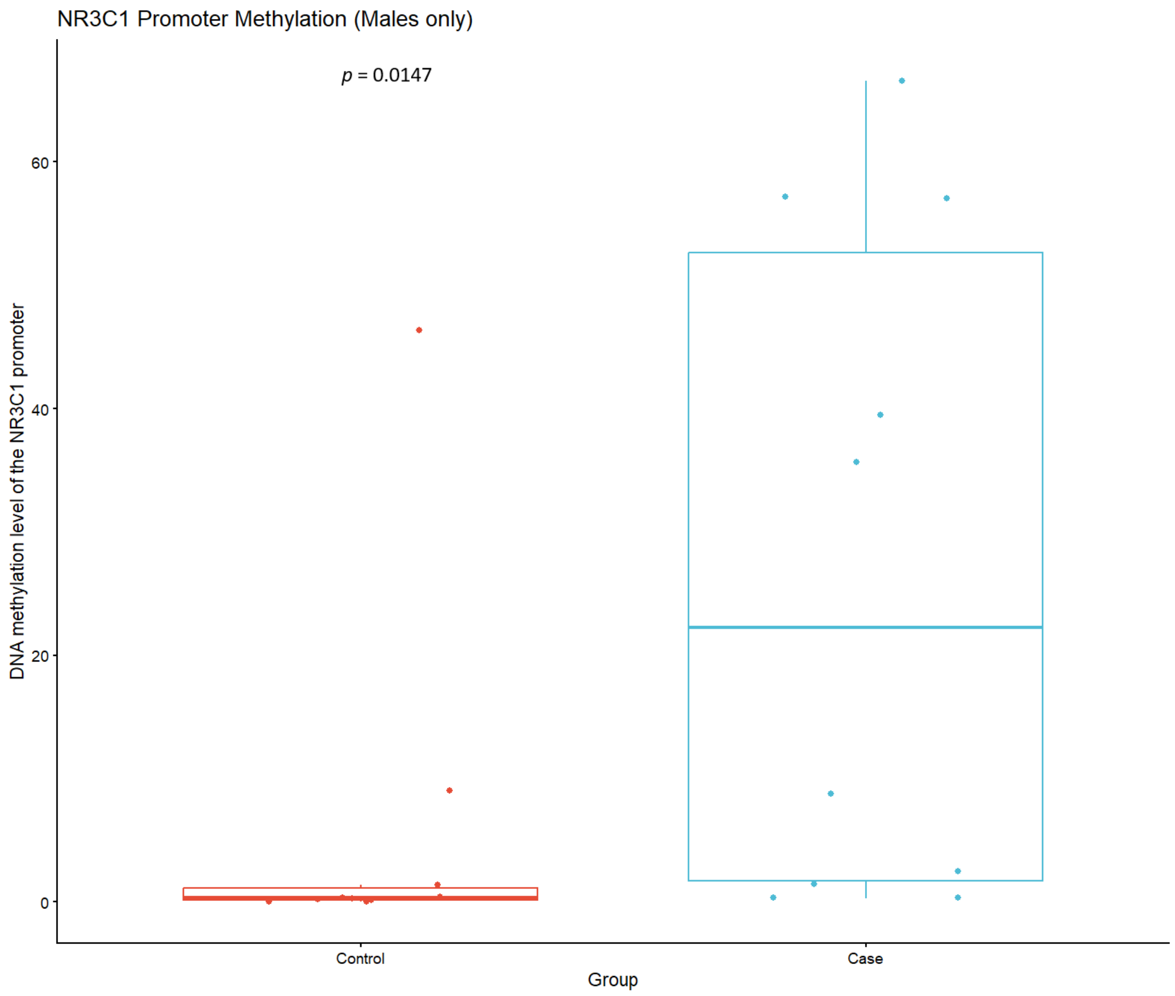

Supplementary Figure S2. ***NR3C1* promoter methylation in dlPFC (males only).** Box plot showing the percentage of DNA methylation at the *NR3C1* exon 1F promoter region in dlPFC (Brodmann area 9) samples from suicide cases and non-psychiatric controls restricted to males (n ≈ 10 per group).

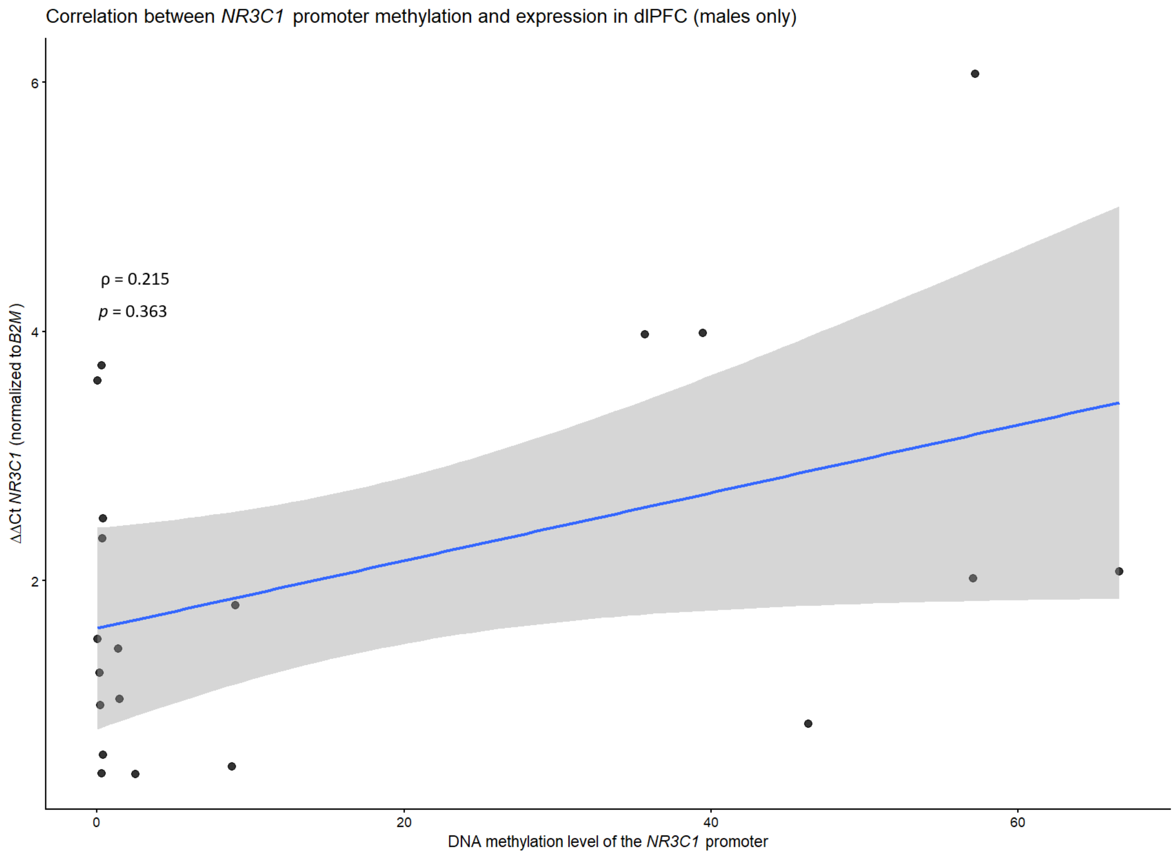

Supplementary Figure S3. **Correlation between *NR3C1* promoter methylation and expression in dlPFC (males only).** Scatter plot depicting the association between *NR3C1* exon 1F promoter methylation (%) and *NR3C1* mRNA expression in dlPFC (Brodmann area 9) restricted to males (n = 20).
